# A Visit from Captain Kidd

**Published:** 1885-12

**Authors:** 


### A VISIT FROM CAPTAIN KIDD.

In an illustrated article on Gardiner's Island, in the December *Century*, George Parsons Lathrop writes as follows, regarding an unexpected visit from the notorious pirate: "Lord John Gardiner one June evening observed a mysterious sloop with six guns riding at anchor off the Island. It was Kidd's last vessel, the *Antonio*. This Lord John was a large, hearty man, who lived generously, was 'clever' to the Indians and squaws, and had so much ability in affairs that, although he married four times and spent a great deal of money, he portioned off his daughters handsomely and left a large estate at his death. He was not a person to be scared by a mysterious armed sloop; so, after she had lain in sight two days without making any sign, he put off in a boat, to board her and inquire what she was. As he came up over the side, Captain Kidd—till then unknown to him—received him with the traditional politeness of a thriving desperado, and asked after the health of himself and family. Then, in answer to Lord John's inquiries, he said that he was on his way to Lord Bellomont at Boston: would Gardiner do him the favor to carry two negro boys and one negro girl ashore, to be kept there until he returned or sent an order for them? Gardiner consented, and went back to the island. The nexting morning Kidd resumed intercourse by sending ashore a request that Gardiner should come on board at once, and bring six sheep with him. This was rather forcing the acquaintance, Gardiner may have thought; but he complied. Thereupon Kidd promptly ripened acquaintance into intimacy, and asked him if he could spare a barrel of cider. Lord John once more proved neighborly, and found that he *could* spare the cider, sending two of his men ashore to fetch it. While waiting for their return, Kidd got out from his cargo two 'pieces' of damaged Bengal muslin,—a rare and valued fabric in its pristine state,—which he put into a bag and requested Gardiner to take as a present to his wife. It is likely enough that the captain, seeing in hearty Lord John a capacity for such things, produced some of his fifty-shilling rum, or three-hundred-pound Madeira to be tasted. Something, at any rate, warmed him up to increased generosity, for 'in about a quarter of an hour' he presented the Lord of the Isle with some muslin for his own use. When the men came back with the barrel of cider, he gave them four pieces of gold for their trouble. Furthermore, after getting ready to sail, he offered to pay for the cider; but Gardiner protested that he was sufficiently rewarded by the present to his wife. They parted at last; and Kidd, gallantly firing a salute of four guns, stood for Block Island.

"His purpose in lingering in these waters was to get rid of his suspicious freight before going to Boston. During his stay near the island two New York sloops took off part of his cargo; and three days later he returned from Block Island in company with another nefarious sloop, which relieved him of chests containing plate and gold and other goods. This time Kidd again sent for Gardiner and committed to his charge a chest, a box of gold, a bundle of quilts, and four bales of goods. The box of gold, as Gardiner afterwards solemnly deposed, was destined by Kidd for Lord Bellomont. All the treasure and merchandise was buried in some swampy land near Cherry Harbor, beside Home Pond, within a mile of the manor-house, to be kept for Kidd or his order.

"'If I call for it and it is gone,' Kidd declared to Lord John, 'I will take your head or your son's.'"

